# Oral Mucosa Models to Evaluate Drug Permeability

**DOI:** 10.3390/pharmaceutics15051559

**Published:** 2023-05-22

**Authors:** Elena Mazzinelli, Ilaria Favuzzi, Alessandro Arcovito, Raffaella Castagnola, Giorgia Fratocchi, Alvaro Mordente, Giuseppina Nocca

**Affiliations:** 1 Dipartimento di Scienze Biotecnologiche di Base, Cliniche Intensivologiche e Perioperatorie, Università Cattolica del Sacro Cuore, Largo Francesco Vito 1, 00168 Roma, Italy; 2 Fondazione Policlinico Universitario “A. Gemelli”, IRCCS, Largo Agostino Gemelli 8, 00168 Roma, Italy; 3 UOC Odontoiatria Generale e Ortodonzia, Dipartimento Scienze dell’Invecchiamento, Neurologiche, Ortopediche e della Testa Collo, Fondazione Policlinico Universitario “A. Gemelli”, IRCCS, Largo Agostino Gemelli 8, 00168 Rome, Italy; 4 Dipartimento di Testa-Collo e Organi di Senso, Università Cattolica del Sacro Cuore, Largo Agostino Gemelli 8, 00168 Rome, Italy

**Keywords:** ex vivo oral mucosa model, in vitro oral mucosa models, oral mucosa equivalents, permeability of drugs

## Abstract

Due to its numerous advantages, such as excellent drug accessibility, rapid absorption, and bypass of first-pass metabolism, the route of drug administration that involves crossing the oral mucosa is highly favored. As a result, there is significant interest in investigating the permeability of drugs through this region. The purpose of this review is to describe the various ex vivo and in vitro models used to study the permeability of conveyed and non-conveyed drugs through the oral mucosa, with a focus on the most effective models. Currently, there is a growing need for standardized models of this mucosa that can be used for developing new drug delivery systems. Oral Mucosa Equivalents (OMEs) may provide a promising future perspective as they are capable of overcoming limitations present in many existing models.

## 1. Introduction

The oral route is the most popular drug delivery method among patients because it is less expensive, easier to use, and does not require specialized medical assistance. The main drawback is the reduced bioavailability of the drug due to its quick breakdown in the gastrointestinal tract (GIT) and first-pass liver metabolism. For these reasons, alternative transmucosal administration methods, such as those through the vaginal, ocular, nasal, buccal, and oral mucosae, have received increased attention in recent studies [1,2,3,4]. Specifically, the oral mucosa is distinguished by superior drug accessibility, quick absorption due to relatively high blood flow, a robust epithelium, bypass of first-pass metabolism, and less exposure of medicines to the gastrointestinal environment [5,6].

**Figure 1:**
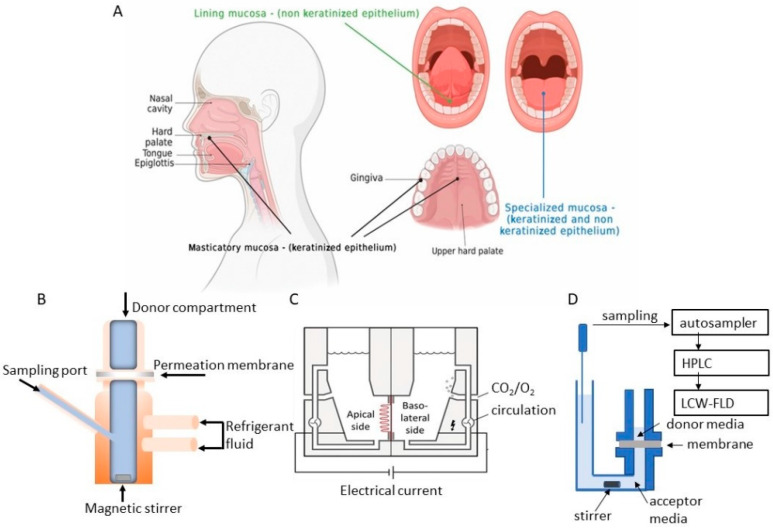
(**Panel A**) Localization of different areas of the oral mucosa where the tissues were extracted to test the permeability of the drugs. (**Panel B**) Franz Diffusion Cell. (**Panel C**) Ussing Chamber. (**Panel D**) Kerski Chamber. Created with BioRender.com (accessed on 17 November 2022).

The structure of the oral mucosa is characterized by an outermost layer with stratified squamous epithelium, an intermediate layer with a basement membrane, and finally, an innermost layer that is composed of connective tissue forming the lamina propria and submucosa [7]. Different studies have shown that the outermost layer is the main barrier to drug diffusion, while the underlying layers are relatively permeable [8,9] (see Figure 1 Panel A).

The permeability of the mucosa is also influenced by the presence of membrane-coating granules rich in keratohyalin, which are found both in the keratinized and in the nonkeratinized epithelium [4]. In the former, they are organized in lamellae, and in the nonkeratinized epithelium, they are rounded and filled with amorphous material. Due to these differences, the permeability of the nonkeratinized epithelium to many compounds increases [4].

Therefore, according to the specificity of this route of administration and more detailed knowledge of the composition of the oral mucosa, a considerable advancement has been made during the last decades in therapeutic drug delivery systems (DDS) designed to sustain a novel approach for the treatment of a wide number of disorders [10].

DDSs are all those procedures that employ biology, chemistry, and engineering principles to safely transport pharmaceutical compounds within the body as required to produce the desired therapeutic effect [11,12].

These systems have a number of benefits, including an increased pharmacological efficacy, decreased adverse effects, improved water solubility and chemical stability of the active ingredients, and regulated drug release [13,14].

Popular examples of DDS are represented by nanocarrier systems, including nanoparticles, liposomes, and micelles [15], which, depending on the therapeutic goal and on the needs of each individual, may be selected.

Drug delivery through oral mucosa can be potentially carried out in any area of the oral cavity, but sublingual and buccal routes are the preferred routes for systemic delivery, as these areas are characterized by higher permeability due to the structure of these specialized tissues (see Table 1). Moreover, to facilitate this process, specific physicochemical properties of the drug may be optimized, such as molecular weight, hydrophilicity/hydrophobicity ratio and ionization [16,17].

The sublingual route is characterized by a nonkeratinized epithelium that is thinner and more vascularized with respect to the buccal one, which makes it even more permeable; therefore, it is a feasible site in acute pathological manifestations when a rapid onset of therapeutic effect is highly desired.

On the other hand, this area is also characterized by high salivation, which, together with the movement of the tongue, washes away the drug; thus, the contact time with the absorption sites is reduced, preventing knowledge of the quantity of that active compound that really penetrates.

The advantages of the buccal route of administration involve the smoother and relatively immobile surface and the greater tolerability towards allergens. The main disadvantage, also in this case, is related to salivary flow. This issue, shared by both buccal and sublingual administration methods, may be overcome by ad hoc delivery systems. In this regard, various DDSs, including fibers, strips, inserts, implants, films, gels, wafers, sprays, and microparticles, have been developed based on biomaterials with mucoadhesive properties, such as chitosan, alginate, cellulose derivatives, and carbomers [18,19,20]. As a paradigmatic example, Jin et al. created a mucoadhesive patch for the targeted administration of methotrexate in oral cancer, enabling site specificity, programmed drug release, and improved patient compliance [21].

Topical treatment of the oral mucosa can also be carried out through both the nonkeratinized and keratinized epithelium (the last present in the gums and palate), even if the latter cannot be used for systemic treatment because it is less permeable [22].

Although many studies have been performed concerning oral mucosal drug delivery, few compounds are currently available on the market that are specifically formulated for this route of administration. Certainly, one of the main reasons is the lack of standardized oral mucosal systems in vivo and in vitro, which would allow us to predict and quantify desired effects and potential pitfalls [23,24].

Moreover, in recent years, the use of in vivo models has been discouraged and significantly reduced due to the development of ex vivo oral mucosa models (derived from animal mucosae) or in vitro models (obtained utilizing cell cultures). However, a standardized system reproducing oral mucosa properties, allowing a rational synthesis of pharmaceutical formulations resistant to salivary flow, movement of the tongue, and chewing, is highly desirable and not yet available [17].

Nevertheless, numerous ex vivo and in vitro models are currently used depending on the investigations being carried out [24], with each model offering certain advantages and disadvantages for evaluating the permeability of the drugs.

This narrative review’s objective is to discuss the state-of-the-art oral mucosal models—which are not commercially available—used to assess the permeability of drugs, paying special attention to the more ambitious vehiculated forms. In this regard, efforts were made to choose the more appropriate models based on the particular effect that needed to be determined.

## 2. Ex Vivo Models

Ex vivo samples derived from animal buccal tissues are often used as models for human buccal epithelium, as tissues of human origin are rare.

Among animals, oral mucosa is mostly obtained from pigs, rats, hamsters, rabbits, dogs, and primates [24]. All these models present specific limits mainly related to characteristics such as thickness and keratinization, even if they are acceptable for studying the trans-buccal absorption of selected drugs. Obviously, it is important to consider these differences during analysis to properly scale up these results for humans (Table 2).

It is evident that using different animals to obtain oral mucosa samples is the first reason for the lack of a standardized ex vivo model; nevertheless, other factors also hinder this aim. In fact, when mucosa derived from the same animal species is used, the following factors can also hinder standardization: different cell culture conditions, a limited amount of tissue from the cheek, and the intrinsic instability of oral mucosa due to the stress that the animal undergoes before slaughter [29]. Regardless, all these variants, both in the origin and in the preparation of the tissues for the models and in the experimental techniques, prevent the standardization of ex vivo permeation studies [30,31].

Consequently, it is very difficult to obtain adequate quality control regarding the evaluation of permeability and cell viability, the latter of which is essential for maintaining the barrier ability in mucosa models. Cell viability is generally determined by 3-(4,5-dimethylthiazol-2-yl)-2,5-diphenyltetrazolium bromide assay (MTT) at the beginning of the permeation tests (which take several hours) and not at the end of the tests [31,32].

To solve this problem, in a recent paper [33], the authors utilized an MTT assay to evaluate the mucosa viability before and after permeability tests, thus comparing the cell viability of five different mucosal models extracted and preserved under different modalities and conditions. In particular, the media utilized were phosphate buffer solution (PBS), Kreb’s bicarbonate Ringer’s solution (KRP), KRP+ 1% fetal bovine serum (FBS), and KRP+ 1% FBS in a CO_2_ atmosphere. The mucosa ex vivo models were rats, rabbits, dogs, pigs, and humans. The separation of the epithelium from the underlying connective tissue by heat treatment resulted in an epithelial thickness of approximately 500 μm without compromising the permeability and integrity characteristics of all different mucosae. Specifically, the authors placed oral mucosa (used in the permeability experiments) in a 6-well plate and cut the sample. A solution of MTT was added to each well, and after 4 h of incubation, the cells were lysed, and the formazan crystals were solubilized by DMSO. Thus, the absorbance at 540 nm was measured in each well, and the viability was determined relative to fresh mucosa, which was assumed to be 100% viable [33].

The obtained results confirmed that mucosae maintained their maximum integrity in KRP at 4 °C for 36 h without using any other protectant. Moreover, the authors reported that in the presence of selected cryoprotectants (20% glycerol and 20% trehalose), the mucosae, which were frozen at −80 °C and thawed at 37 °C, exhibited preserved integrity and biological viability for 21 days. Therefore, this study aimed to identify the experimental conditions to standardize the process of isolating, maintaining, and determining the viability of mucosa and thus improve the accuracy of permeability studies [33].

The permeability of a drug is defined by the coefficient “Log P”, with this parameter being the partition coefficient of the selected molecule between aqueous and lipophilic phases, which are usually water and octanol. [34,35]. Accordingly, Log P is an intrinsic property related to the chemical structure of a drug and its ability to perform hydrophilic or hydrophobic interactions between a nonionized form of the drug and its medium. [34,35].

Moreover, some drugs are ionized at physiological pH; therefore, the distribution coefficient “log D” (diffusion) provides a more precise method to evaluate their permeability [34,35]. This value varies according to the pH of the medium [36,37], and it is always calculated at a specific pH, where the ionization degree and, consequently, the lipophilicity of the drug, is well-known.

The relationship between the degree of ionization and the permeability of drugs has been the topic of numerous studies [38,39,40], and results always showed that absorption depends on the degree of compound ionization.

Moreover, using ex vivo models, different diffusion chambers are commonly used to evaluate the diffusion properties, according to the position of the barriers where the ex vivo tissues are placed: vertical (Franz diffusion chambers) [24] and side-by-side horizontal (Ussing chambers) [41] (see Figure 1 Panel B,C).

The Franz diffusion chamber works in static conditions. It is composed of a donor compartment in which the solution containing the drug is applied to the apical surface of the mucosa. The temperature is maintained at 37 °C [42] and samples are removed after 180 min. Due to the static conditions, obstacles can arise that hinder drug absorption. Some authors have proposed the use of cosolvents, such as methanol or ethanol, but these compounds alter mucosal permeability [24].

An improvement in the Franz chamber was achieved through the introduction of a medium flow that mimics salivary flux, helping to maintain absorption conditions [43].

Another method to evaluate the transport of many compounds through the buccal mucosa is the modified Ussing chamber [44,45,46]. It consists of two half chambers separated by biological tissue. Cellular vitality is maintained by oxygen flux [47].

To quantify drug permeation, both chambers can be equipped with UV/VIS spectroscopy or HPLC [48], but both of these techniques show limited analytical specificity and sensitivity. Moreover, both these chambers have limited possibility of sampling automation and standardization [32,49].

Despite these limitations, these experimental tools allow us to measure the drug permeability in the mucosa; thus, a large number of works have also used these devices to evaluate the effect of drug carriers on this parameter (Table 3).

A specific use of porcine buccal tissue inside the Franz-diffusion chamber was designed by Serpe et al. [79]. In this study, the authors demonstrate ex vivo the effect of salivary washout on drugs delivered to the oral cavity using coated microneedles. In particular, they observed that salivary flow increases permeation with respect to the control. In the conclusions, the authors state that future studies are necessary to optimize in vitro salivary flow simulation to develop a more suitable in vitro permeation model. Recently, a new permeation model was indeed proposed and validated by Majid et al. [31,80]. The authors utilized a novel automatized vertical Kerski diffusion chamber (see Figure 1 Panel D) with a total volume of 12 mL and with controlled environmental conditions. This chamber has a horizontal part to maintain the tissues that are always immersed in the cell culture medium.

A further innovative aspect that was introduced by the group of Majid et al. was coupling the Kerski chamber to Hanson Research AutoPlusTM and HTS PAL which allow the authors to quantify a specific drug via liquid chromatography mass/mass spectrometry. Within the 60-min permeation period, Hanson Research AutoPlus^TM^ performed nine fully automated samplings and transferred the samples through MultiFill^TM^ into LC vials for drug quantitation.

Sample preparation was performed automatically by the HTS PAL autosampler using Chronos XT software. Cell proliferation and cytotoxicity were evaluated by appropriate kits (Cell Counting Kit-8) [31,80].

The obtained results showed a high level of standardization and automation with guideline-compliant relative errors. The authors concluded that their results are aligned with the physiological–clinical conditions of therapeutic doses. The automation of the procedure represents a significant improvement in the standardization of permeability testing [31,80].

Furthermore, to apply oral mucosa ex vivo models to permeability studies, different novel aspects must be considered when a drug is administered through delivery systems or without them [81].

Indeed, in the first situation, under specific environmental conditions, the diffusion and partition coefficients are intrinsic properties of the drug. On the other hand, when the drug is administered, the entire formulation must be evaluated. The predominant concern in preparing mucosal drug delivery systems is the concentration of active compounds that are released from the delivery system. Indeed, if the drug is tightly bound to functional moieties present in the delivery system, its bioavailability may be significantly reduced [81]. Therefore, in a specific formulation mediated by a drug delivery construct for the oral cavity and, specifically, for the oral mucosa, there is a delicate balance between the stability of the entire DDS and its ability to release the active drug in a specific time and spatial window.

Therefore, different permeability assessment procedures are used in the presence of delivery systems that cannot enter the cells (such as nanofibers, implants, etc.) or delivery systems able to cross the mucosal barrier (nanoparticles, etc.). In both cases, an important application of the ex vivo models is the comparison of the drug’s traditional administration formulation with its vehiculated form, thus allowing the optimization of the new pharmacological formulations [81].

In this context, Bashya and coworkers [68] used porcine mucosa models to determine the permeability of hydrophobic ion-pairing (HIP)-nano complexes loaded with insulin, utilizing a vertical static Franz diffusion cell with an effective diffusion area of 0.79 cm^2^. Similarly, in a recent paper, Sharifi and coworkers [70] evaluated the permeability of diclofenac sodium vehiculated with nanofibers of different materials in comparison with a traditional gel formulation, using fresh sheep buccal mucosa in Franz diffusion cells.

Regarding the evaluation of the permeability of drugs loaded inside delivery systems that cannot cross mucosa, a porcine buccal mucosa model was used in a recent paper [67] to determine if nicotine present in nicotine replacement therapy (NRT) formulations—for oromucosal administration—can penetrate throughout the buccal mucosa and surpass the epithelial barrier. In this paper, the author used α- lactalbumin/polyethylene oxide nanofibers. The same model was used by Eleftheriadis and coworkers [69] to determine the permeation profiles of lidocaine hydrochloride, combined with the permeation enhancer l-menthol, deposited onto a mucoadhesive film by inkjet printing.

In both studies, the obtained results were encouraging and intriguing, and the permeability was also evaluated using TR146 cells.

Finally, porcine oral mucosa was also used to evaluate the ability of nanoparticles loaded with metronidazole and inserted in a hydroxyethyl cellulose gel to cross the oral mucosa [71].

Since oral transmucosal administration of a drug is not the only transmucosal route of administration, an interesting application of ex vivo mucosal models is the determination of the best route of application for a pharmacological preparation. An example of this interesting application was reported by Silva-Abreu [72]. In this work, the authors synthesized PLGA-PEG nanoparticles loaded with pioglitazone (PGZ-NPs) and evaluated their ability to penetrate different ex vivo mucosal systems using buccal, sublingual, nasal, and intestinal specimens.

The obtained results showed that PGZ-NPs with a dimension of approximately 160 nm exhibit a high permeability in all mucous membranes, even if the best performances were found in the intestinal mucosa. Apart from these interesting results, the authors highlighted the main parameters that should be evaluated to determine the permeability of a drug carried by NPs and how they must be interpreted [72].

In particular, the authors used Franz diffusion cells to measure PGZ permeation per unit area (μg/cm^2^) at established time intervals. Measurements were performed in all types of mucosae, and the retained PGZ was measured by HPLC on the extracted mucosae at different time points [72].

To optimize the comparison between the different mucosal samples, the authors evaluated the cumulative amount of PGZ (μg) permeated through the mucosa and plotted it against time (h). All subsequent values are obtained graphically and proven by the related equations [72].

### Limits of Ex Vivo Oral Mucosa Models

Based on the scientific literature mentioned above, it can be concluded that ex vivo models are optimal for both the analysis of drug permeability and for reducing the number of in vivo experiments; however, due to atherogenicity related to tissue origin and preparation, these models cannot completely replace in vivo tests, even if more recent publications are making progress in standardizing and automating many experimental procedures [31,72,80].

## 3. In Vitro Models

Alternative to ex vivo models are in vitro models obtained utilizing human cells cultured in vitro. This model became necessary because animal and human buccal epithelia exhibit different properties [82].

**Figure 2:**
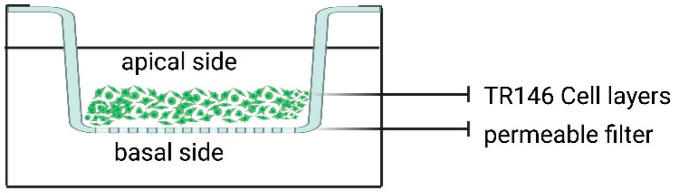
TR146 in vitro model Created with BioRender.com (accessed on 10 March 2023).

In 1995, filter-grown TR146 human cells, a cell line originating from a human buccal carcinoma [83], were used to produce an oral epithelium model in vitro to investigate cell permeability to β-adrenoceptor antagonists [84] (see Figure 2).

In this work, the authors optimized the growth conditions of TR146 and showed that cells can, alternatively, form a monolayer or a multilayer (thickness 40 µm in 21 days) [84]. In many experimental papers, the integrity of the cellular layers was further verified by permeability tests, such as the paracellular flux of ions (by the transepithelial/transendothelial electrical resistance—TEER—technique [17,84,85,86,87,88,89,90,91,92,93]) or tracer flux assays [84,85,86,87,88,89,90,91,92,93]_._ Through TEER, it is possible to determine the barrier integrity of a cell layer by measuring its electrical resistance. This technique, which is based on impedance spectroscopy, consists of devices with two sets of voltmeters, allowing for the TEER to be continuously analyzed and providing information about the barrier properties of the membrane model [94]. In contrast, the tracer flux assay involves using fluorescent or radiolabeled probes that can cross the cell layers. The most frequently used compounds are fluorescein, mannitol, sucrose, etc. [23], for hydrophilic molecules.

The results obtained in TEER studies showed that the permeability of the TR146 in vitro model [150–200 Ωcm^2^] is higher than that of the healthy epithelium, which is due to their derivation from a carcinoma. Additionally, experiments with tracer flux assays [86] showed that compounds such as mannitol or testosterone crossed the TR146 barrier faster than human mucosa. These results are probably due to an alternative set of differentiation markers present on TR146 with respect to the normal oral epithelium [95]. Moreover, Jacobsen et al. 1995 [84] also reported that tight junctions are not present in TR146 cells and other authors [91] reported that zona occludens is also poorly represented in this cellular model. Nonetheless, TR146 cells are used to study the drug’s permeability in the vehiculated form (Table 4), and in vitro models obtained utilizing tumour cell lines are only partly able to represent the barrier properties of the normal oral mucosa [23]. Therefore, utilizing TR146 in permeability studies is generally limited to evaluating variations of this parameter, i.e., between a drug administered in vehiculated form versus a non-vehiculated one (Table 4), or to evaluating the effects of enhancer compounds on drug permeability [87].

In 2019, Chen et al. [74] used a TR146 cell culture to perform permeation tests of carvedilol delivered via self-assembled liposomes and core/shell fibers using water-soluble bioadhesive polymers.

Experiments were also conducted with porcine buccal mucosa. The obtained results showed that both delivery systems favored the penetration of the drug into the cell. Another interesting application of TR146 in permeability was described in a very recent paper [74].

Another interesting application of TR146 in permeability was described in a further recent paper [99]. In this work, the authors used TR146 cells to determine if nanoparticle composition influences cellular uptake. For this purpose, the following formulations were prepared: (i) solid lipid nanoparticles with palmitic acid; and (ii) nanostructured lipid carriers with palmitic acid and oleic acid in different ratios.

In vitro results showed that the composition of the particles influenced permeability. The liquid lipid oleic acid increased the cellular uptake capacity without changing the underlying uptake mechanism. Regarding the route of the uptake, it was demonstrated that both types of nanoparticles can penetrate inside the cells due to caveolin-mediated endocytosis, as shown by particle localization in the endoplasmic reticulum [99].

Due to the relative simplicity of the TR146 model, it is also possible to apply this system to study the mechanisms related to the permeability of molecules. In fact, in a very recent paper [97], the authors not only demonstrated that the permeation of hyaluronic acid across TR146 depends on its relative mass but also that hyaluronic acid can enhance oral barrier integrity due to the stimulation of genes involved in the formation of tight junctions.

### Limits of In Vitro Oral Mucosa Models

Based on the above reports, it can therefore be concluded that in vitro models are optimal for comparing the permeability of different preparations of the same drug or for clarifying the mechanisms related to permeability; however, due to their tumour origin, the methods cannot fully replace in vivo models.

## 4. Oral Mucosa Equivalents

To bypass the limits of previously described in vitro models, oral mucosal equivalents (OMEs) have been recently developed [100]; as a result, drug delivery systems can be better studied. These OMEs are composed of normal oral keratinocytes (NOK) cultured on top of normal oral fibroblasts (NOF). NOKs are characterized by poor longevity and interindividual variability; therefore, Jennings et al. [101] developed a model based on commercial TERT2-immortalized oral keratinocytes (FNB6). The histology and expression of the structural markers are similar to those observed in normal oral mucosa and in previously used OMEs.

OMEs are used to study host-pathogen interactions, [102], cancer biology, [103], tissue regeneration [104], and drug permeability [105]. When OMEs are applied to evaluate drug delivery systems, it is necessary to verify their barrier properties and their viability. The aim of a recent paper [105] was precisely to verify the possibility of exploiting these properties of OMEs for the delivery of corticosteroids. The OMEs were constructed as reported previously [101], and their vitality was evaluated by Alamar Blue assay. The presence of tight junctions, desmosomes, and hemidesmosomes was confirmed by microscopy observation, while the barrier properties were measured by TEER and tracer flux assays, using fluorescently labelled dextran (molecular mass range from 3 to 70 kDa). The OME model was exposed to corticosteroid formulations and incubated for up to 24 h. Thus, the media present in the lower well of the transwell chamber were collected and the OME was washed, weighed, and disaggregated. Finally, the corticosteroid concentration was determined by HPLC. The obtained results showed that the permeation of corticosteroids into the OMEs was unaffected by the delivery form. This study confirmed that OMEs are suitable models for evaluating both toxicity and drug delivery.

Oral mucosa models can also involve gingival mucosa, and this is particularly important because the availability of human keratinocytes and fibroblasts is low owing to the size of donor biopsies.

Buskermolen et al. solved this problem in a paper published in 2016 [106] by developing a human gingival equivalent constructed from cell lines, both keratinocytes, and fibroblasts immortalized with TERT. The gingival equivalent was characterized by immunohistochemical staining for cell proliferation, epithelial differentiation, and basement membrane production. All these parameters were like those of human gingiva. The experiments conducted in this paper confirmed that the gingival equivalent may be a good alternative to the animal model in studying new therapeutic formulations [106].

Nevertheless, all the models previously reported do not consider the presence of mucus. Thus, due to the role played in mucosa protection, it is very important to consider mucus during the design of innovative transmucosal drug delivery systems.

To the best of our knowledge, there are no mucus-producing in vitro mucosal models; however, 2D in vitro mucus-containing buccal models have been developed by Teubl et al. [91], who placed a mucus layer on the two-dimensional cell culture TR 146. A similar model was utilized by Marxen et al. [107] to evaluate the mucus effects on the permeability of small molecules, such as propanol, caffeine, nicotine, and mannitol. The results showed that the permeability of propanol and caffeine was decreased by the presence of mucin; in contrast, nicotine and mannitol permeability was not affected [107].

Ployon et al. [108] investigated the role of MUC1 in the binding of MUC5B to oral mucosae. In this work, the authors did not evaluate the effect of the mucus on the drug’s permeation, but they validated a 2D model able to produce mucus that could also be used for this purpose.

## 5. Conclusions and Future Perspectives for Oral Mucosa Models

As previously reported, the use of the oral mucosa as a drug administration route necessitates the development of specific drug delivery systems that, in addition to biocompatibility and biodegradability, are also mucoadhesive or bioadhesive, pleasant in flavor, and exhibit a palatable texture to improve patient compliance [10,11,12]. To characterize these delivery systems, it is necessary to verify the ability of the active ingredient to penetrate the oral mucosa. Carrying out in vivo studies involves several disadvantages, including the high cost and the ethical problem associated with animal sacrifice. [109]. This last problem is less strict in the European Union, as the use of animals to test drugs is still permitted, contrary to the evaluation of oral care products (Regulation EC No 1223/2009). However, Directive 2010/63/EU on the protection of animals used for scientific purposes (14G00036) clearly indicates that the number of animals used to evaluate the safety and efficacy of a drug or medical device must meet the following 3Rs [109,110,111]:

(1) Replacement: the animal species utilized in the study are those with the lowest neurological development;
(2) Reduction: the study utilized the minimum number of animals;
(3) Refinement: the method is optimized to reduce animal suffering during the execution of the procedures.

For these reasons, over the years, many research laboratories have developed models of the oral mucosa capable of reproducing the structure and function of native mucosal tissues that permit the following:

(1) Reduce the number of animals sacrificed;
(2) Reduce experimental costs;
(3) Focus on specific issues related to drug delivery due to the absence of in vivo complexity [111].

However, there is still no standardized model for determining the oral mucosa suitable for all necessary situations when developing a new drug delivery system. Ex vivo models are extremely useful in examining the passage of the drug within the mucosa, but it is extremely difficult to make comparisons with non vehiculated drugs. [23,24].

In contrast, the single-cell line in vitro models exhibit strong reproducibility, but their permeability values are different compared to those of human mucosa. Nevertheless, the models are intensively used because the drug permeability can be easily compared using different administration forms. Therefore, the selection of one model rather than the other is linked to the type of experiment being performed [23,24].

OMEs show the potential for a wider application in the near future as they manage to overcome some limitations present in both previous models. However, significant work is still needed to define a functional multilayered in vitro organization that can mimic the in vivo situation—that is, a comprehensively modeled salivary flux and can predict the performance of different formulations in an experimental setting that simulates the clinical one. 

## Figures and Tables

**Table 1:** Different types of mucosae in the oral cavity.

Type of Mucosa	Localization	Characteristics
**Masticatory mucosa** (25% of the oral cavity’s surface)	- Gingival region; - Hard palate.	- Thick and keratinized epithelium; - Strong adhesion to the lamina propria; - In some regions, there is no submucosa presence.
**Lining mucosa** (60% of the oral cavity’s surface)	- Inner surface of the lips and cheeks; - Lower face of the tongue; - Sublingual sulcus; - Soft palate.	- Nonkeratinized epithelium with different thickness depending on the region; - Elastic surface; - More permeable than the other types of oral mucosa.

**Table 2:** Characteristics of different oral mucosa models.

Animals	Kind of Epithelium	Advantages	Disadvantages
Rats	Keratinized		Different permeability [25]
Hamsters	Keratinized		Different permeability [25]
Rabbits	Non-keratinized or keratinized	The permeability is like the human mucosa	The amount of mucosa is significantly reduced [26]
Dogs	non-keratinized		The epithelium is thinner with respect to human one, thus the permeability is different [26]
Monkeys	non-keratinized		The epithelium is thinner with respect to human one, thus the permeability is different [26]
Pigs	Non-keratinized or keratinized	The permeability values are similar with respect to human mucosa [27,28]	

**Table 3:** Application of ex vivo models in permeability studies.

Active Compounds	Vehicle	Excision Animal Tissues (Ex Vivo Models)
5-Aminolevulinic acid	No	porcine buccal mucosa [50]
5-aminolevulinic acid	Chitosan based mucoadhesive films	Pig buccal (Cheek) mucosa [51]
Quercetin	Core-Shell Composite Microparticles	Porcine sublingual mucosae [52]
Acyclovir	Gels	porcine oral mucosa [53]
Betahistine dihydrochloride	mucoadhesive tablets	Camel buccal mucosa [54]
Lidocaine	Gel	pig palatal mucosa [55]
5-Fluorouracil	Tablets	porcine buccal mucosa [56]
Diazepan	No	Porcine buccal mucosa [57]
Risperidone	mucoadhesive gel formulation	Porcine buccal mucosa [58]
Cannabidiol	No	Pig buccal tissues [59]
Isoniazid	micelles	Porcine buccal mucosa [60]
Nicotine	enhancer	Porcine buccal mucosa [61]
Diazepan	No	porcine buccal mucosa [62]
Lamotrigine	No	Porcine buccal tissue [63]
Ondansetron	Film	Porcine oral mucosa [64]
Metronidazole	Gel	Porcine buccal mucosa [65]
Insulin	Fibers	porcine cheek tissues [66]
Isoniazid	micelles	Human buccal mucosa [60]
Nicotine	Nanofibers	Porcine buccal mucosa [67]
Insulin	hydrophobic ion-pairing (HIP)-nano complexes	Porcine buccal mucosa [68]
Ketoprofen and lidocaine	Film	Porcine buccal mucosa [69]
Diclofenac	nanofibers	sheep buccal mucosa [70]
metronidazole	hydroxyethyl cellulose-based gel containing metronidazole-loaded solid lipid nanoparticles	Porcine oral mucosa [71]
Pioglitazone	PLGA-PEG Nanoparticles	different ex vivo mucosal systems: buccal, sublingual, nasal, and intestinal [72]
fluorescence-labeled nanoparticles to investigate penetration efficiency to oral mucosal tissues	ester-based core-multishell nanoparticles	porcine masticatory and lining mucosa [73]
Carvedilol	self-assembled liposomes and core/shell fibers	Pig buccal mucosa [74]
DOPA 3,4-dihydroxy-D-phenylalanine	PLGA NPs	porcine buccal tissue [75]
Peptide	multi-layered nanofiber-on-foam-on-film	porcine buccal mucosa [76]
furosemide	hollow mesoporous silica nanoparticles	Porcine buccal mucosa [77]
Zolmitriptan and Etodolac	film comprising chitosan, sodium alginate, and ethyl cellulose	rabbit buccal mucosae [78]

**Table 4:** Application of TR 146 in permeability study.

Active Compounds	Vehicle
Furosemide	Mucoadhesive buccal films based on a graft co-polymer—A mucin-retentive hydrogel scaffold [96]
Furosenimide	Hollow mesoporous silica nanoparticles [78]
leu-enkephalin	No vehicles, the goal was the comparison between TR146 and human buccal epithelium [86]
Testosterone	No vehicle [84]
Metformin	Bio adhesive chitosan discs [87]
Carvedilol,	Self-assembled liposomes and core/shell fibers [74]
low molecular weight Hyaluronic Acid <100 kDa and >500 kDa	No vehicle, the goal was to study the dependence of permeability by molecular weight hyaluronic acid and tight junction modulation in human buccal TR146 [97]
Zolmitriptan and Etodolac	Film comprising chitosan, sodium alginate, and ethyl cellulose [78]
Peptides	Oral guar films entrapping peptide-containing chitosan microparticles TR146 [98]

## Data Availability

Not applicable.
